# Team-based assessment of professional behavior in medical students

**Published:** 2014-07

**Authors:** HOJAT RAEE, MITRA AMINI, AMENEH MOMEN NASAB, ABDOLRASOUL MALEK POUR, MOHAMMAD MORAD JAFARI

**Affiliations:** 1 Medical School, Shiraz University of Medical Sciences, Shiraz, Iran;; 2 Quality improvement in Clinical Education Research Center, Shiraz University of Medical Sciences, Shiraz, Iran;; 3 Health information and management school, Shiraz University of Medical Sciences, Shiraz, Iran

**Keywords:** Assessment, Students, Professional, Behavior

## Abstract

**Introduction**: Self and peer assessment provides important information about the individual’s performance and behavior in all aspects of their professional environment work. The aim of this study is to evaluate the professional behavior and performance in medical students in the form of team based assessment.

**Methods**: In a cross-sectional study, 100 medical students in the 7^th^ year of education were randomly selected and enrolled; for each student five questionnaires were filled out, including one self-assessment, two peer assessments and two residents assessment. The scoring system of the questionnaires was based on seven point Likert scale.  After filling out the questions in the questionnaire, numerical data and written comments provided to the students were collected, analyzed and discussed. Internal consistency (Cronbach’s alpha) of the questionnaires was assessed. A p<0.05 was considered as significant level.

**Results**: Internal consistency was acceptable (Cronbach’s alpha 0.83). Interviews revealed that the majority of students and assessors interviewed found the method acceptable. The range of scores was 1-6 (Mean±SD=4.39±0.57) for the residents' assessment, 2-6 (Mean±SD= 4.49±0.53) for peer assessment, and 3-7 (Mean±SD=5.04±0.32) for self-assessment. There was a significant difference between self assessment and other methods of assessment.

**Conclusions**: This study demonstrates that a team-based assessment is an acceptable and feasible method for peer and self-assessment of medical students’ learning in a clinical clerkship, and has some advantages over traditional assessment methods. Further studies are needed to focus on the strengths and weaknesses.

## Introduction

Performance assessment of medical students is essential in the clinical environment. In a traditional model of education in North America, in a ‘preceptor-based’ arrangement, one student is assigned to one physician for a defined period of time and this physician is responsible for daily assessment of the student’s clinical performance based on the professional behaviors. This model relies on repeated interactions over time between the student and preceptor and takes no account of the behaviors of other members of his/her team (1).

One of the important features of contemporary medical education is assessment to ensure quality in training programs, motivate students, and direct what they learn (2). Multi-Source Feedback (MSF) assessment provides important information from individual’s performance and behavior in all aspects of their professional environment work (3). The method improves self-evaluation skills (4), enhances communication skills (5), addresses complaints of assessor-bias (6), and provides a valuable feedback from those best qualified to assess certain behaviors (7).

MSF has been widely used in residency programms and for physicians in practice (5, 8, 9), but MSF had not been used enough in assessment of undergraduate medical students. Rees and Shepherd (10) have also evaluated professionalism in medical students, using a 360-degree assessment model.

Medical professionalism is defined as a set of values, attitudes, and behaviors that results in serving the interests of patients and society before one’s own (11-14). Professionalism remains as one of the most difficult core content areas in medical education. So many studies have evaluated professional development of medical students using peer assessment methods (15-18). Anne et al. revealed that peer assessment can be a powerful tool to assess and encourage formation of professional behaviors, particularly the interpersonal dimensions (19).

The aim of this study is to design and implement an assessment method including team-based multi-source method in which clinical and professional performance of medical students would be evaluated by themselves, peer students and residents.

## Methods

In a cross-sectional study, 100 students in the 7th year of medical education were randomly selected. For each student, five questionnaires were filled out, including one self-assessment, two peer assessments and two resident assessments. 

The assessment questionnaire was developed after reviewing the existing assessment items focusing on nine capabilities:

1. How to communicate with other professional medical personnel
2. Ability of communication with patients
3. Amount of ability in evaluation of patients
4. How to deal with people who have low social levels
5. Amount of professionalism in practice
6. Consultation with other medical personnel
7. How to follow up the patients in the emergency department
8. Measurement of the needs of learning more
9. Peer learning or teaching the students with lower grades

The questionnaire was extracted from the study of Crossiey (20) and its validity and reliability was confirmed by the faculty of Shiraz University of Medical Sciences (Table 1). The authors have reviewed and discussed items from these sources several times to draw an agreed list of statements describing the spectrum of desired performance of medical students.

Different groups of medical students completed the questionnaire and were also asked about their opinion on how many forms should be completed on a single student. Complexity of the team-based setting allowed the students to initiate the process of assessment by asking an assessor to complete the questionnaire. The questions in the questionnaire form were scored from 1 to 7 on a Likert scale and the students were informed that score 1 in each question means the performance similar to a first year student and score 7 means that the performance is like a professional physician.

Internal consistency (Cronbach’s alpha) was calculated using SPSS 14 (SPSS Inc, Chicago, IL, USA). Descriptive statistical reports were prepared by calculating mean and SD of the whole questionnaire and each item. Differences among the self assessment scores, peer assessment and resident assessment were measured by ANOVA test. P<0.01 was considered as significant.

## Results

In all, 500 forms for 100 students were filled out, including 100 self-assessments, 200 peer assessments and 200 resident assessments. Students completed peer assessment forms on each other (200), self-assessment forms for themselves (100), and resident assessment (200). Fifty residents completed the questionnaire for all the involved students. Table 2 describes the number of individuals participating from each assessor group, the reliability of the item responses for each assessment form, and the number of forms completed. The internal consistency of each assessment form was acceptable, ranging from Cronbach’s alpha 0.83-0.91, excluding the administrator form which was completed by only one assessor.

**Table 1:** Assessment form for professional behavior of last year medical student

**Dear Student** **This questionnaire is designed for self-assessment of your professional behavior, please score yourself in each item (score 1, the lowest number is when you act like an amateur student and score 6 when you act like a professional student) ** **Male □ Female □**							
**Score** **Items**	**1** **(Performance such as first year student)**	**2**	**3**	**4**	**5**	**6**	**7** **(Performance such as professional physician)**
**When I work with professional physician team**							
**When I make relation with the patients**							
**When I evaluate my patient**							
**When I am faced with cultural apposite individuals of hygiene environments**							
**When I consider professional behavior in my work**							
**When I have consultation with other physians**							
**When I am involved with emergency patients**							
**When I think about my clinical experiences to determine my learning needs**							
**When I teach to my classmates or students at lower grades**							

Score ranges resulting from the residents' assessment was 1-6 (Mean Score= 3.9); the peer assessment 2-6 (Mean Score= 4.3); and self-assessment 3-7 (Mean Score= 5.1). The mean scores for self-assessment, peer assessment and resident assessment for each item in the questionnaire are shown in Table 3. There was a significant correlation among self-assessment, peer assessment, and residents assessment (p<0.05) but there was no significant correlation between different items in the questionnaires.

All 100 students at the last academic year submitted the required number of signed assessment forms on time; no student had any scores more than two out of 2 or less and no student failed this element of his/her assessment.

Students reported working with a median of nine different residents over the 8 weeks. Residents assessed a median number of 24 final year students during the academic year (range 1–56). An assessment form was completed on average after a student worked 2.2 days with a resident (assessment range 1-6). 

Interviews were performed with 2 attending physicians and two chief residents. Although the proportion of assessors and students attending the interview was low, the experts’ opinion was obtained by informal feedback throughout the year. There was a general consensus that having assessors other than physicians was a good idea. The attending physicians also appreciated the immediacy of giving feedback right after a teaching session, and appeared to prefer this to the online evaluation system in which they would sometimes complete an assessment weeks or months after the student had worked with them. Of the chief residents interviewed, one believed the method of assessment made sense, while the others did not like the method in general, preferring the former preceptor-based model. Both stated that current assessment forms should be revised. 

**Table 2:** Results of assessment of professional behaviors for the last year medical students

** **	**Assessed medical students**	**Peer reviewers**	**Residents**
**Degree of education**	Last year Student	Last year Student	Chief residents
**Frequency **	100	200	200
**Age (Mean± SD)**	24.1± 1.51	24.7±2.1	30.2±3.4
**Score range**	3-7	2-6	1-6
**Mean score **	5.04±0.32	4.49±0.53	4.39±0.57
**Internal consistency (Cronbach’s alpha)**	0.83		

Interviews with students suggested that the majority liked being assessed by more than one attending physician or residents. They stated that they liked the immediacy of the assessment, as opposed to systems in other clerkships that would offer feedback only after that clerkship was over. The assessment items were considered to be straight forward and easy to understand, although students reported valuing written comments. Assessments of residents and patients were particularly highly valued by students. Students stated that patient assessments were sometimes difficult to obtain, or awkward to ask for, and some felt they were giving the impression of caring more for the patient because they were going to ask for an assessment to be completed. Students were also pleased that they could have some control in choosing the timing of their assessments. Some reported being able to temper negative feedback from one team member in the light of positive feedback from others. Some students reported their concern about having to strategize to get the right number of assessment forms completed and reported trouble in tracking down assessors, or in remembering to have the forms signed. One student stated ‘having to ask people how they think you’re doing is now the scariest thing about the surgery clerkship.’ Another reported: ‘I have to be on my best behavior all the time now, as everyone I work with is assessing me!’

**Table 3:** The mean and standard deviation of responses to each item of the questionnaire

**Score** **Items**	**Self-assessment** ** Mean±SD **	**Peer assessment** ** Mean±SD **	**Residents assessment** ** Mean±SD **
**When I work with professional physician team**	4.66±0.84	4.43±0.77	4.62±0.76
**When I make relation with the patients**	4.7±0.63	4.43±0.96	4.47±0.77
**When I evaluate my patient**	5.56±0.50	4.74±0.75	4.51±0.76
**When I am faced with cultural apposite individuals of hygiene environments**	4.97±0.64	4.24±0.98	4.42±0.92
**When I consider professional behavior in my work**	5.6±0.51	4.55±0.87	4.27±1.01
**When I have consultation with other physicians**	4.92±0.80	4.71±0.84	4.19±1.03
**When I am involved with emergency patients**	5.45±0.50	4.38±0.88	4.30±0.91
**When I think about my clinical experiences to determine my learning needs**	4.74±0.61	4.43±0.82	4.34±0.83
**When I teach to my classmates or students at lower grades**	4.74±0.71	4.51±0.95	4.36±0.98

## Discussion

This study demonstrates that a team-based model of assessment is a valuable form of assessment for medical students learning in a clinical clerkship. By engaging assessors and students in development of the assessment tool, and by providing instruction and advice on how the tool should be used, we designed an acceptable assessment form based on the opinion on multiple groups of assessors and students working in complex clinical environments at multiple sites. We also succeeded in engaging members of the healthcare team who have not traditionally been involved in the assessment of medical students. This method of assessment gives a voice to non-physician team members not traditionally involved in medical student assessment, i.e. nurses, patients, administrators, and peers.

Previously, a physician might have solicited the opinion of these members informally before completing a student’s assessment; now these members of the team can comment directly on aspects of student performance that they observe. Other authors have shown that non-physicians are able to evaluate the communication skills and humanism of physicians in training and practice (21, 22). We believe that using a team-based method of assessment also encourages students to interact more with members of the healthcare team and pay attention to how their behavior is perceived by their peers, coming much closer to a true 360-degree assessment of medical students than other studies which have claimed the same (23). Demonstrating the importance of collaboration with other team members is an important message to send to medical students in training (24, 25). We believe that soliciting the opinions of peers, patients and administrators is important; many of the comments provided by these groups are related to areas of performance not usually observed by a supervising physician. In our experience, the information provided to students using this method is mainly formative in nature. No student failed the assessment, and most of the comments provided to them were positive and encouraging. As each student was observed by up to 4 observers, and no single assessor had the power to fail a student, we believe this method of assessment is essentially a series of low stakes ‘mini-assessments’ which are cumulated into a final report containing all of the feedback received. It should be noted here that this method of assessment was only one part of a larger assessment plan employed in addition to other traditional summative methods including a multiple-choice examination and an OSCE. We believe that assessment is made more robust using a variety of tools to measure the student performance, and that this method may be one way of achieving the ‘frequent look’ system of assessment proposed by Ricketts and Bligh (26).

For students with deficient performance, information given by multiple observers allows a more complete picture of their performance to be obtained, to help guide discussions with the student after the conclusion of the clerkship. Having a one page summary of a student’s performance including detailed information from all methods of assessment used and including comments from all observers proved helpful in our experience when making decisions on academic promotion and advancement during the months after the clerkship had finished. This method of assessment appeared to be relatively labor-intensive compared to an automated online assessment system. Most of the ‘work’ of scheduling assessments is done by students and assessors completing an assessment form shortly after the students have worked in a real clinical context. We believe that this method of assessment offers the ability to provide immediate feedback on recently observed student behavior, a major advantage over the other systems which we previously employed. The financial cost of printing, binding and scanning assessment books is also acceptable in our institution. Having the assessment logbook retained by students for the full 6 weeks of the clerkship has some limitations, as the logbook is vulnerable to loss or damage and there is a potential risk of ‘forward-feeding’ if an assessor looks at other assessment forms completed before completing his/her own form (27).

Physicians reported their comfort with the assessment tool and with the fact that students were being evaluated by others in the patient-care team. We had anticipated some resistance from physicians who had traditionally been the ‘source of truth’ in student assessment relinquishing this role to the team, but this did not turn out to be the case (28).

Using a method in which students are required to initiate or ask for an assessment was initially challenging, but it was gradually accepted by both learners and assessors. This study has a number of limitations, the most important of which is that we couldn’t include nurses and patients as assessors. Therefore we made a number of changes to the assessment method in the second year of its use, such as allowing the students to select their own ward nurse assessors, allowing physicians to record how long they had spent with a student, designing the new form for patients’ assessment, and allocating more space on the forms for written comments. This study demonstrates that team-based assessment can indeed be implemented and accepted in a clinical clerkship. We plan to continue to study this method of assessment to determine its strengths and weaknesses, to further examine its reliability and validity, and to compare this method with more traditional methods of assessment used elsewhere in our school.

## Conclusions

This study demonstrates that a team-based model of assessment based on the principles of MSF is a feasible and valuable form of assessment for medical students learning in a clinical clerkship; also, it has some advantages over traditional preceptor-based assessment. Further studies are needed to demonstrate the strengths and weaknesses of this novel assessment technique.
